# Carnosine Synthase (*Ts*ATPGD) Alleviates Lipid Peroxidation Under Transcriptional Control by an *Nfe2*-like Gene in *Tridacna Squamosa*

**DOI:** 10.3390/antiox13111351

**Published:** 2024-11-04

**Authors:** Zhuo Yang, Nai-Kei Wong, Fan Mao, Siwei Wu, Wenjie Yi, Ziniu Yu, Yang Zhang

**Affiliations:** 1 Key Laboratory of Breeding Biotechnology and Sustainable Aquaculture, Key Laboratory of Tropical Marine Bio-Resources and Ecology and Guangdong Provincial Key Laboratory of Applied Marine Biology, South China Sea Institute of Oceanology, Chinese Academy of Sciences, Guangzhou 510301, China; yangz@idsse.ac.cn (Z.Y.); maofan@scsio.ac.cn (F.M.); wusiwei21@mails.ucas.edu.cn (S.W.); yiwenjie20@mails.ucas.ac.cn (W.Y.); carlzyu@scsio.ac.cn (Z.Y.); 2 Sanya National Marine Ecosystem Research Station, Tropical Marine Biological Research Station in Hainan, Key Laboratory of Tropical Marine Biotechnology of Hainan Province, Chinese Academy of Sciences, Sanya 572000, China; 3 Department of Pharmacology, Shantou University Medical College, Shantou 515041, China; wongnk@stu.edu.cn

**Keywords:** *Tridacna squamosa*, Nfe2l gene, carnosine synthase (ATPGD), lipid peroxidation, *L*-carnosine

## Abstract

As an important mollusk in reef ecosystems, *Tridacna squamosa* forms pro-survival symbiotic relationships that hinge on an exquisite redox equilibrium between the host and the photosynthetic symbiont, zooxanthellae. The exact regulatory mechanisms thereof remain poorly understood. In this study, a novel Nfe2-like transcription factor in *T. squamosa* was identified and characterized with respect to its antioxidant and cytoprotective roles. Gene structure and phylogenetic analysis reveal that *T. squamosa* possesses a single transcription factor *Ts*Nfe2l in contrast to mammalian Nfe2l1 (Nrf1) and Nfe2l2 (Nrf2), belonging to protein members of the bZIP-NFE2 subfamily and functionally resembling the mammalian Nfe2l1. A conserved bZIP domain permits its binding to the antioxidant response element (ARE) in vitro and in HEK293T cells. Further analyses such as promoter prediction suggest that *Ts*Nfe2l target genes engage mainly in the regulation of multiple enzymes involved in antioxidation and allied pathways. Notably, *Ts*Nfe2l transcriptionally upregulates carnosine synthase (*Ts*ATPGD), which subsequently produces *L*-carnosine abundantly to shield cells from oxidative damage. Moreover, the blockage of *Ts*Nfe2l nucleic acid binding reduced the expression of *Ts*ATPGD and *L*-carnosine content in the gill, resulting in elevated lipid peroxidation. Collectively, our findings establish novel molecular insight into *Ts*Nfe2l as a critical regulator of redox homeostasis in *T. squamosa* through carnosine synthesis.

## 1. Introduction

The giant clam *Tridacnidae squamosa* is a symbiotic bivalve species endemic to the Indo-Pacific. Like corals, the majority of its nutrients are supplied by photosynthetic zooxanthellae of the genus *Symbiodinium* [1,2,3], which is housed almost exclusively in the giant clam’s iridescent mantel. Disruption to this exquisite symbiotic link is mechanistically analogous to the pathophysiology seen in coral bleaching. *T. squamosa* is thus increasingly being recognized as an ecologically and biologically significant organism worthy of illuminating the intricate processes of symbiosis as well as ecologically relevant to marine biodiversity in the face of climate change.

A multitude of regulatory pathways are involved in *Tridacnidae squamosa*–zooxanthellae symbiosis, which sustains such processes as oxidation resistance, calcium deposition, nitrogen metabolism, and tissue development [4,5,6]. During photosynthesis, however, the symbiont zooxanthellae not only supply energy to the host but also create some collateral antioxidant demands. When extrinsic conditions for photosynthesis turn unfavorable, an impaired photosynthetic electron transport chain in zooxanthellae starts to produce excessive amounts of reactive oxygen species (ROS) [7]. Sustained accumulation ROS in host tissues destroys *Tridacnidae squamosa*’s antioxidant capacity, leading to organismal injury, bleaching, or eventual death. In particular, bleaching is a phenomenon wherein the zooxanthellae become abnormally rejected from the host, rendering clam tissues white and incapable of photosynthesis, as characterized by severe lipid peroxidation [6,8]. A critical gap remains as to how *Tridacnidae squamosa* successfully manages oxidative stress in marine habitats.

The internal homeostasis of nearly all forms of aerobic life requires antioxidant defense mechanisms to cope with extrinsic assaults [9,10]. In mammals, this pivotal function is governed by bZIP-NFE2 family member proteins and their binding target, the antioxidant response element (ARE) [9,11,12]. Within this diverse family, NFE2, Nfe2l1, Nfe2l2, and Nfe2l3 are known to regulate cellular responses conducive to homeostasis [13,14]. Mammalian Nfe2l1 and Nfe2l2 have been extensively studied for their prominent roles in antioxidant defense in the contexts of human health and disease, such as cancer, aging, and metabolic disorders [15,16,17]. As one facet of cellular oxidative stress, lipid peroxidation is commonly initiated by excessive ROS [18], reactive nitrogen species (RNS) [19], and other reactive metabolites disruptive to cellular redox balance, leading to the secondary generation of reactive lipid species (RLS) and harmful oxidation footprints such as malondialdehyde (MDA). Lipid peroxidation, in turn, promotes cellular dysfunction and cell death (notably ferroptosis) to set in motion aging pathologies [20] and other diseases [21]. Genes such as glutathione peroxidases (GPX) and glutathione *S* transferase (GST) are well-known targets of the bZIP-NFE2L protein family, which alleviate lipid peroxidation directly or indirectly [22]. This highlights the critical roles of bZIP-NFE2L family members in combating lipid peroxidation. Still, little is known about how these Nfe2l-related pathways are regulated or utilized in the contexts of cross-species symbiosis. The ability of *T. squamosa* to maintain redox homeostasis in the presence of heterologous photosynthetic cells suggests its potential for complex defense against lipid peroxidation, with bZIP-NFE2L family members being evidently implicated. However, there are few known studies about antioxidant defense mechanisms in such symbiotic marine organisms.

The roles of bZIP-NFE2L family members are clearly underexamined in the *T. squamosa* antioxidant response, especially in the context of lipid peroxidation, which warrants some devoted investigation. From an evolutionary perspective, the coordinated antioxidant responses in lower animals as natural reductionist models present unique opportunities to scrutinize gene structure and function, which may be masked by biological complexity in higher animals [23,24,25]. In this study, through a combinatorial approach of omics data analyses and molecular biology, we addressed this gap by cloning an Nfe2-like gene in *T. squamosa* and advanced a detailed illustration of its cytoprotective function against lipid peroxidation mediated by a downstream effector gene, carnosine synthase (*Ts*ATPGD). The findings thus presented should aid the construction of a theoretical framework for the systematic conservation and breeding of *T. squamosa*.

## 2. Materials and Methods

### 2.1. Experimental Animals and Samples Collection

Adult individuals of *Tridacna squamosa* were obtained from the giant clam breeding base of South China Sea Institute of Oceanology, Chinese Academy of Sciences in Sanya, Hainan Province, China. The animals were kept in an indoor aquarium with circulating artificial seawater, maintained at 27 °C under a dark–light cycle (12 h: 12 h). Following acclimation for two weeks, different tissues including hemolymph, outer mantle, inner mantle, muscle, foot, heart, and gills were collected from three individuals at 6 h of the light phase. Total RNA was then extracted by means of the Trizol reagent according to the manufacturer’s instructions. DNA was extracted from the outer mantle by using the HiPure Mollusc DNA kit (Magen, Guangzhou, China). The remaining gill tissue was divided into 5 portions of an equal weight for tissue culture. Both RNA and DNA were stored at −80 °C for later use. This study was conducted in accordance with the ethical regulations set forth by the Animal Care and Use Committee of Life Sciences, South China Sea Institute of the Chinese Academy of Sciences, Guangzhou, China.

### 2.2. Analysis of TsNfe2l Protein Sequence

Human Nfe2l1 (NP_003195.1), Nfe2l2 (NP_006155.2), and Nfe2l3 (NP_004280.5) protein sequences were used as query entries, while all protein sequences predicted from the *T. squamosa* genome were used as subject entries in BlastP analysis to obtain a *Ts*Nfe2l reference sequence ID. After nucleic acid and protein sequences were extracted by using TBtool, BlastN/P was run with the NCBI NR database to verify integrity. The same method was used for the identification of *Ts*MafK (query sequence NP_002351.1).

All sequences of the phylogenetic trees were obtained from NCBI. MAFFT was used for sequence alignment. IQTree was used for phylogenetic tree construction. The MEME function of MEME Suite was used to find *Ts*Nfe2l proteins’ motifs [26]. Functional domains of *Ts*ATPGD and *Ts*MafK were predicted by using NCBI’s Batch CD-Search. The results above were summarized using TBtool [27].

For the analysis of interspecific gene families, all genomic data, except for those of *T. squamosa*, were retrieved from NCBI. Gene family members were obtained from NCBI. The results were summarized accordingly by using TBtool v2.119.

### 2.3. Molecular Cloning of TsNfe2l and TsMafK

The primers used for the PCR amplification of ORF (open reading frame) of the two genes are as shown in Table 1. All primers used were commercially synthesized (Tianyi Huiyuan, Beijing, China). RNA (about 1 µg) prepared from the *T. squamosa* outer mantle was reverse-transcribed into cDNA by using a PrimeScript^TM^ 1st Strand cDNA Synthesis Kit (TaKaRa, Osaka, Japan). The cDNA was diluted to about 100 ng/µL and used as a template for PCR by using TaKaRa Ex Taq (TaKaRa, Osaka, Japan), with the following conditions according to the manufacturer’s protocol: 95 °C (3 min); 95 °C (15 s), 60 °C (15 s), and 72 °C (30 s to 3 min) for 35 cycles; and 72 °C (5 min). Amplification products were purified with a HiPure Gel Pure DNA Mini Kit (Magen, Guangzhou, China) and then stored at −20 °C until use.

### 2.4. Electrophoretic Mobility Shift Assay (EMSA) on TsNfe2l Activation

The CDS (coding sequence) regions of *Ts*Nfe2lMBR (*Ts*Nfe2l multiple binding region) and *Ts*MafK were inserted between the EcoRI and XhoI sites of the pGEX-4T-1 vector by using the MonClone^TM^ Single Assembly Cloning Mix (Monad, Hangzhou, China). The primers used are as shown in Table 1. On confirmation of construct validity by sequencing, the plasmid was then used to transform the *E. coli* competent strain BL3. The transformants were cultured to the exponential phase in LB (Luria-Bertani) broth at room temperature. Then, the culture temperature was swapped to 16 °C and followed by induction with IPTG (0.5 mM) for 16 h to produce recombinant GST fusion proteins. The harvested proteins were purified on an affinity chromatography column packed with GST Sefinose^TM^ Resin (BBI Life Sciences, Shanghai, China), dialyzed, and stored at −80 °C until use.

The ARE probe was generated by PCR by using the pARE-Luc (Beyotime, Haimen, China) as a template, which was then purified following verification via agarose gel electrophoresis. The probe for the promoter core element (*Ts*ARE) of the *Ts*Nfe2l target gene (*Ts*ATPGD) was synthesized by annealing a single-stranded nucleic acid. The primers and single-stranded nucleic acid used are as shown in Table 1. The aforementioned probes contain two *Ts*Nfe2l binding sites.

EMSA was performed by using the Molecular Probes fluorescence-based EMSA kit (Invitrogen, Carlsbad, CA, USA), according to the manufacturer’s instructions. Images for the EMSA results were acquired by the ChemiScope 6000 (Clinx Science Instruments Shanghai, Shanghai, China).

### 2.5. Dual-Luciferase Reporter Assay

Coding sequences of the recombinant fusion protein hNfe2l2ΔMBR-*Ts*Nfe2lMBR (containing a.a. 1-466 of hNfe2l2 and a.a. 714-885 of *Ts*Nfe2l), the recombinant fusion protein *Ts*Nfe2lΔMBR-hNfe2l2MBR (containing a.a. 467–605 of hNfe2l2 and a.a. 1–713 of *Ts*Nfe2l), the recombinant fusion protein hNfe2l2Neh4 + 5-*Ts*Nfe2lMBR (containing a.a. 109–219 of hNfe2l2 and a.a. 714–885 of *Ts*Nfe2l), and *Ts*Nfe2l were inserted between EcoRI and XhoI sites of pcDNA3.1-V5-HIS-A by using MonClone^TM^ Single Assembly Cloning Mix (Monad, Beijing, China). The hNfe2l2 fragment (NM_006164.5 as an insert sequence; Vigene, Shanghai, China) of the fusion protein was amplified by using pENTER-hNfe2l2 as a template. A segment spanning the ninth upstream base of the core element and the transcription start site of the predicted promoters of *Ts*ATPGD was inserted between the Xhol and HindIII sites of pGL3-Basic by using the abovementioned kit. pRL-TK had previously been prepared in the laboratory, and pARE-Luc was procured as described above.

HEK293T cells were maintained in DMEM supplemented with 100 U/mL penicillin-streptomycin and 10% FBS at 37 °C and 5% CO_2_. Prior to the experiments, the cells were seeded into 48-well plates in the same culture medium. Polyethylenimine linear (FuShen, Shanghai, China) was used for transfection, following the manufacturer’s instructions. After 48 h of recovery, cells were transfected with 10 ng pRL-TK, 100 ng reporter plasmid, a certain amount of the coding plasmids, and pcDNA3.1-V5-His-A to make up a total amount of 510 ng plasmids per well.

After 48 h post-transfection, luminescence signals were detected by using the Dual-Luciferase^®^ Reporter Assay System (Promega, Fitchburg, MA, USA) and Infinite M200 PRO (Tecan, Grödig, Austria) following the manufacturer’s instructions. Statistical analysis on all assay results was performed with GraphPad 8. For multiple groups of data, a one-way ANOVA test was performed, followed by intergroup comparisons. For two groups of data, an unpaired *t*-test was performed, with significant difference being determined at *p* < 0.05.

### 2.6. Real-Time qPCR

About 100 ng/µL cDNA from different tissues was used as templates to perform real-time qPCR by using the LightCycler^®^ 480 II (Roche, Basel, Switzerland), according to methods for MonAmp^TM^ SYBR^®^ Green qPCR Mix (Monad, Wuhan, China). The same types of tissues collected from different individuals constituted a set of replicates. *TsGAPDH* was used as a reference gene. The target *T. squamosa* gene sequences in qPCR were identified from the genome using BlastN/P and extracted with TBtool. The primers used in the experiments are as shown in Table 1. Statistical analysis on gene expression was performed with GraphPad 8. Heatmaps were drawn by using TBtool, with the results being normalized according to the tissue types.

### 2.7. Prediction of TsNfe2l Target Genes

According to the *T. squamosa* genome, 2000 bp upstream of all *T. squamosa* mRNAs was assumed to be a promoter region and extracted by using TBtool. The motif prediction for the promoter region was performed with the FIMO function of the MEME Suite, by using hNfe2l2 binding site matrix data from JASPAR (MA0150.1) [28].

GO enrichment of the predicted target genes was performed by TBtool, based on the results from GO genome annotation. Correlation analysis was performed between *Ts*Nfe2l tissue expression and transcriptomic data of different tissues. Venn diagrams for predicted and related genes were drawn with R script v4.2.2 and volcano plots with TBtool v2.119.

### 2.8. Gill Tissue Culture

L-15 was used as the culture medium, which was prepared with sterile artificial seawater, supplemented with 100 U/mL penicillin-streptomycin, 100 µg/mL kanamycin, 2 µg/mL nystatin, 1% gentamicin, and 10% BSA. The abovementioned antibiotics were prepared as disinfectants in sterile artificial seawater, as previously described [29,30,31]. Isolated gill tissue (weighing about 100 mg on average) from each *T. squamosa* individual was dissected into 5 parts. After sterilization with disinfectants for 50 min, the tissue was placed in the culture medium (3 mL) in a 6-well plate, followed by incubation for about 48 h. Two groups were left untreated. Then, ML385 (Cas No.:846557-71-9, MedChemExpress, Monmouth Junction, NJ, USA), a specific inhibitor of Nfe2l2, was added to another two groups to a concentration of 5 µM, while *L-*carnosine was added to the remaining group to yield a final concentration of 50 µg/mL upon adding ML385. Subsequently, a sample from the untreated group and a sample from the group of ML385-treated gill tissues were taken to extract RNA by using Trizol and stored at −80 °C for later use. Each of the remaining three groups of tissues was homogenized with 1 mL ddH_2_O and centrifuged at 12,000× *g* for 10 min to separate the supernatant, while the filtrate was obtained after 3 kDa ultrafiltration.

### 2.9. Assays on Lipid Peroxidation and Carnosine Content

A lipid peroxidation MDA assay kit (Beyotime, Shanghai, China) was used to determine lipid peroxidation levels of the filtrate. The content of *L*-carnosine in the filtrate of the untreated group and ML385 group was detected by LC-ESI-MS. The detection conditions were as follows: chromatographic column: C_18_; liquid phase: A: 0.1% formic acid, (*v*/*v*) B: methanol; sample volume: 30 µL; column temperature: 25 °C; absorbance: 210 nm; program: 0 min, 98% A; 6 min, 98% A; 15 min, 40% A; 15.01 min, 98% A, 20 min, 98% A; 25.00 min, 98% A. The chromatographic peak area and concentration of *L*-carnosine standard products (BBI Life Sciences, Shanghai, China) were used to draw a standard curve, and the content of the *L*-carnosine in each gill tissue was calculated accordingly [32].

## 3. Results

### 3.1. Molecular Cloning and Evolutionary Analysis of TsNfe2l

**Figure 1:**
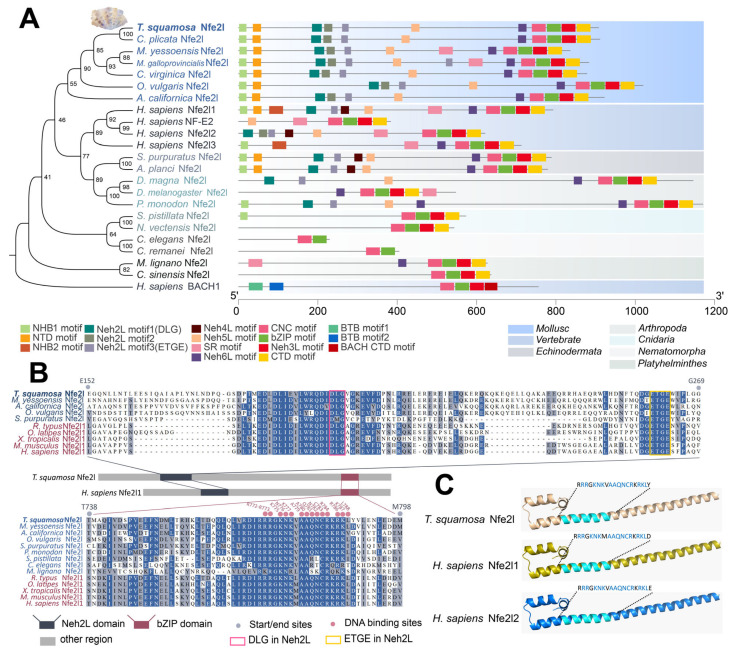
The evolutionary relationship and conserved motif of *Ts*Nfe2l and homologous proteins. (**A**). The ML cladogram and corresponding protein structure between *Ts*Nfe2l and animal homologous proteins of different phyla, as well as members of the human bZIP-NFE2L subfamily. Each square represents a motif, and the motif matrix is generated from the protein sequence in Supplementary Figure S1. *Ts*Nfe2l is shown in bold. The NCBI IDs of human NF-E2 and Nfe2l3 are NP_006154.1 and NP_004280.5, respectively, and other sequence IDs are shown in Supplementary Figure S1. (**B**). The sequence alignment results of the bZIP domain and the Neh2L domain of *Ts*Nfe2l with highly homologous proteins in other species. The DLG- and ETGE-conserved sequences in Neh2L were labeled with square boxes, and key DNA-binding residues in the bZIP domain were labeled with red spheres. (**C**). A comparison of the 3D structure of the bZIP domain DNA-binding regions between *Ts*Nfe2l and Nfe2l1.

A *Ts*Nfe2l homolog was identified in *T. squamosa* through BLASTP searches using human NFE2L subfamily members (Nfe2l1, Nfe2l2, and Nfe2l3) as queries. The predicted *Ts*Nfe2l protein comprises 884 amino acids with an estimated molecular weight of 99.96 kDa and a theoretical isoelectric point of 4.98. Phylogenetic analysis placed *Ts*Nfe2l within the invertebrate NFE2L subfamily, distinct from the vertebrate lineage (Figure 1A, left), and a more detailed evolutionary tree with vertebrate Nfe2l1 and Nfe2l2 showed a similar topology (Figure S1), implying their ancient divergence.

*Ts*Nfe2l possesses an *N*-terminal Neh1-like domain, resembling Nfe2l1 rather than the typical Nfe2l2 structure (Figure 1A, right). Alignment of invertebrate *Ts*Nfe2l homologs with vertebrate Nfe2l1 displayed conserved Neh2L and bZIP domains with high sequence similarity, including a key motif (DLG and ETGE in Neh2L) essential for DNA binding (Figure 1B). Structural analysis of the bZIP domain showed similarity to human Nfe2l1 and Nfe2l2 (Figure 1C), suggesting conserved DNA-binding properties. Comparative genomic analysis indicated that *Ts*Nfe2l is unique to mollusks, with vertebrate genomes typically containing 2–4 NFE2L family members (Figure S2). Furthermore, the mollusk *Ts*Nfe2l homologs exhibit a higher number of exons (9–11 exons) and longer introns compared to their vertebrate counterparts (2–5 exons) (Figure S3), further supporting the independent evolution of Nfe2l after the divergence of the invertebrate–vertebrate NFE2L family.

### 3.2. TsNfe2l Is a Transcription Factor That Recognizes the Typical Sequences of ARE

**Figure 2:**
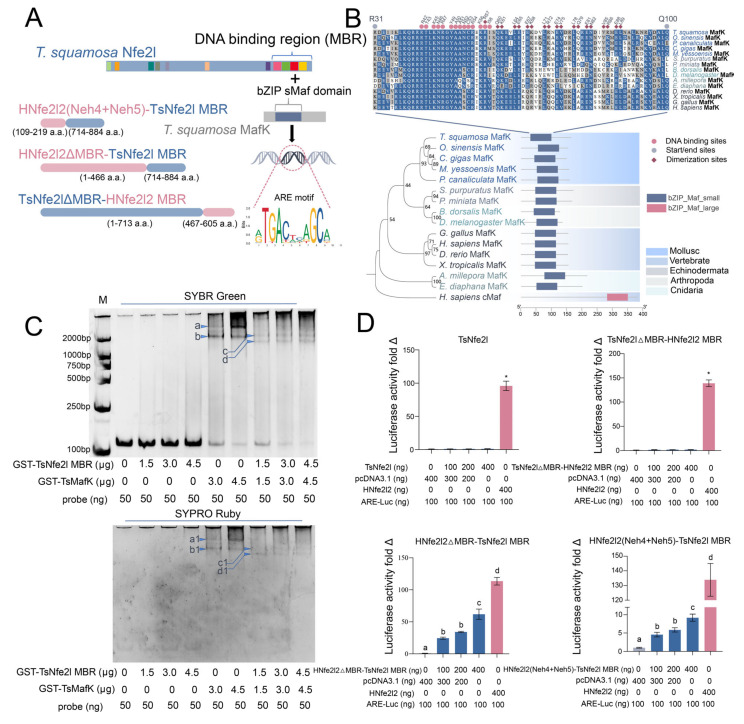
The EMSA and dual-luciferase validation of *Ts*Nfe2l on ARE-binding ability. (**A**). A schematic diagram of the fusion protein structure used for double-luciferase analysis and the binding of *Ts*Nfe2l and *Ts*MafK to ARE after dimerization, in which the motif matrix ID corresponding to ARE is MA0150.1. (**B**). A schematic diagram of ML cladogram and respective domains between *Ts*MafK and homologous proteins of different species. The sequence alignment shows the bZIP sMafK domain, in which the key DNA-binding residues are marked with red spheres and dimerization sites are marked with red diamonds. (**C**). EMSA verifies the binding ability of *Ts*Nfe2l to ARE. M refers to the nucleic acid marker, which contains bands corresponding to 2000 bp, 1000 bp, 750 bp, 500 bp, 250 bp, and 100 bp, respectively, from top to bottom. The probe length is 122bp, including two ARE sites. **a.** The migration bands of a double GST-*Ts*MafK dimer ARE complex. **b.** The migration bands of a single GST-*Ts*MafK dimer ARE complex. **c.** The migration bands of the double GST-*Ts*Nfe2lMBR-GST-*Ts*MafK dimer ARE complex. **d.** The single GST-*Ts*Nfe2lMBR GST-*Ts*MafK dimer ARE complex migration bands; **a1**, **b1**, **c1,** and **d1** are the corresponding protein bands. (**D**). The *Ts*Nfe2l and dual-luciferase analysis results of each fusion protein. The result values were standardized with the mean of the transcription factor (plasmid 0 ng group), expressed as the mean ± S.D., where “*” indicates significant differences with other groups (*p* < 0.01, *n* = 4). Different letters indicate significant differences between groups (*p* < 0.01, *n* = 4).

To elucidate the transcriptional activity of *Ts*Nfe2l, we cloned and expressed *Ts*MafK (Figure 2A), a putative heterodimerization partner of *Ts*Nfe2l [13,15,33]. Phylogenetic analysis confirmed *Ts*MafK as a member of the Maf family, characterized by a conserved bZIP_Maf_small domain (Figure 2B). EMSA analysis demonstrated that both *Ts*MafK and *Ts*Nfe2l-MBR (containing the *Ts*Nfe2l basic region-leucine zipper domain) could bind to the ARE probe, with enhanced binding being observed as the protein concentration increased (Figure 2C). To assess the transcriptional activity of *Ts*Nfe2l in vivo, we employed a dual-luciferase reporter assay using HEK293T cells due to the lack of bivalve cell lines [34]. While *Ts*Nfe2l and *Ts*Nfe2lΔMBR-hNfe2l2MBR failed to activate the ARE reporter, the fusion proteins hNfe2l2ΔMBR-*Ts*Nfe2lMBR and hNfe2l2Neh4 + 5-*Ts*Nfe2lMBR, containing the hNfe2l2 transactivation domain, exhibited robust transcriptional activation. These results indicate that the *Ts*Nfe2l DNA-binding domain can interact with the ARE but lacks an intrinsic transcriptional activation capacity (Figure 2D). It is noteworthy that *Ts*Nfe2lMBR and hNfe2l2 appeared to activate the ARE pathway in 293T cells without the need for the sMaf protein, which might be due to the presence of endogenous sMaf in the background of the 293T cells that facilitated binding. Further sMaf knockout experiments would help to clarify this.

### 3.3. Prediction and Validation of Downstream Genes of TsNfe2l

**Figure 3:**
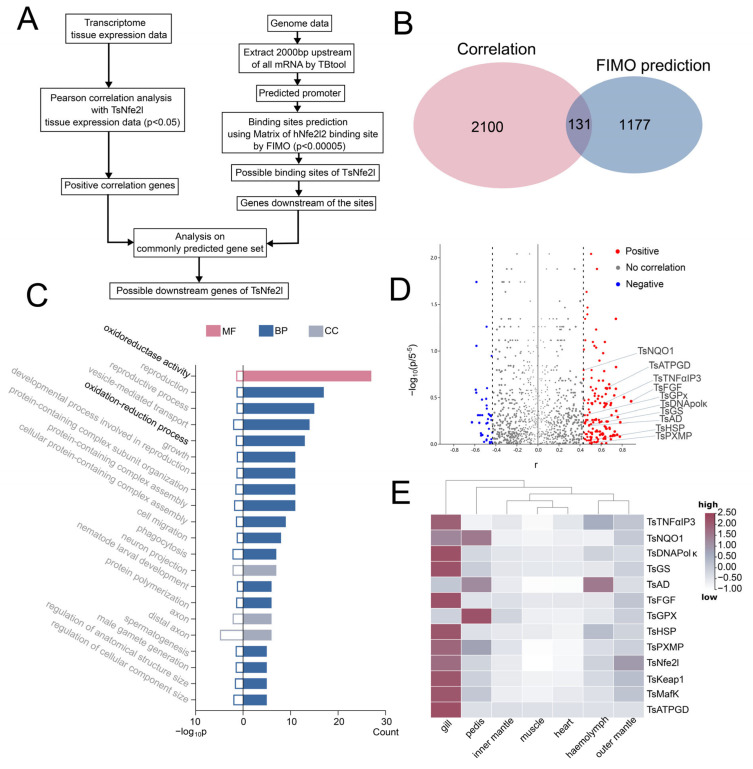
The prediction of the downstream genes of *Ts*Nfe2l. (**A**). A flow chart of the prediction method. (**B**). Venn diagrams of the results of the two prediction methods. (**C**). The GO enrichment analysis results of FIMO prediction results, *p* < 0.05. MF: molecular function, BP: biological process, CC: cellular component. (**D**). A volcano map analysis of two prediction results. The *x*-axis *r* is the correlation coefficient of Pearson’s correlation analysis, and the dotted line indicates *p* = 0.05. The *p* value used in the *y*-axis comes from the *p* value corresponding to each gene in the FIMO analysis results. If a gene corresponds to multiple *p* values (multiple binding sites are predicted in the promoter), the minimum value is taken. (**E**). The tissue expression distribution of *Ts*Nfe2l, *Keap1*, *Ts*MafK, and 10 downstream genes.

To identify potential *Ts*Nfe2l target genes, we predicted *Ts*Nfe2l binding sites within 2000 bp upstream of the *T. squamosa* gene start sites using a known Nfe2l2 binding motif. The overlapped genes with the predicted binding sites and the positively correlated expression with *Ts*Nfe2l were considered potential targets (Figure 3A). In total, 1308 genes with potential binding sites and 2231 genes that significantly positively correlated with *Ts*Nfe2l were identified, among which 131 genes intersected in the *Tridacna* genome (Figure 3B,D). Gene ontology (GO) enrichment analysis of these target genes revealed an overrepresentation of terms related to oxidoreductase activity and oxidation–reduction processes, consistent with the known functions of Nfe2l2. To validate potential target genes, we selected ten genes involved in cell homeostasis and oxidative stress resistance for further analysis. qPCR analysis demonstrated higher expression of these genes in gills compared to other tissues, suggesting a prominent role for the gills in antioxidant defense in *T. squamosa* (Figure 3E).

### 3.4. TsNfe2l Regulates Lipid Peroxidation Through TsATPGD

**Figure 4:**
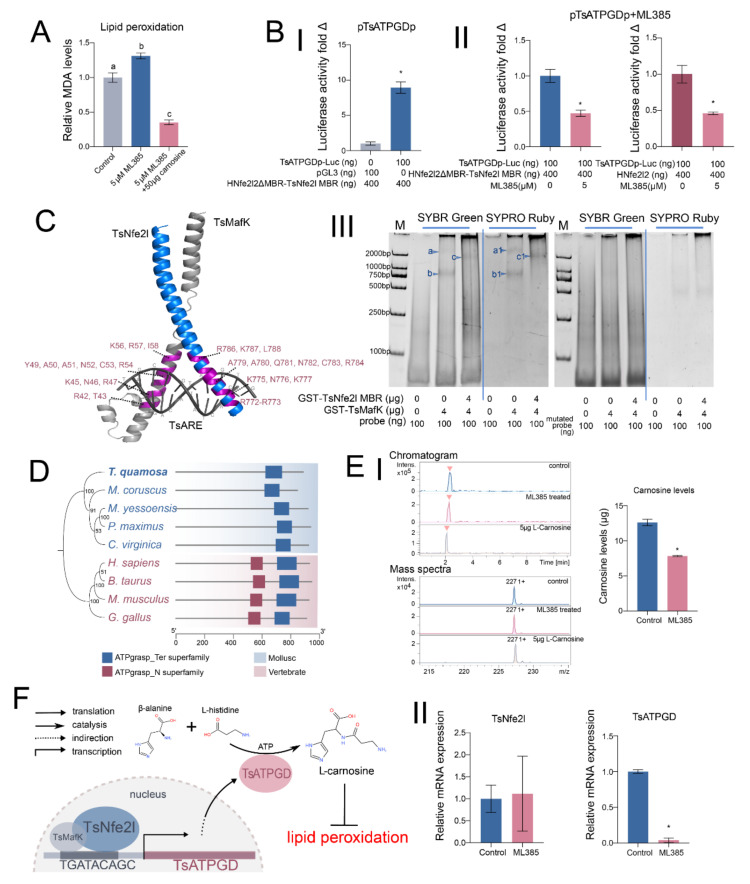
*Ts*Nfe2l inhibits lipid peroxidation by regulating *Ts*ATPGD expression. The bars of different colors in the histogram represent the different treatment groups for each experiment, with the treatment information for each group annotated on the horizontal axis. (**A**) The MDA level in gill tissue before and after ML385 treatment. The control group indicates that no treatment was performed. All values were standardized by the mean value of the control group, expressed as mean ± S.D. Different letters mean that the difference between the groups is significant (*p* < 0.01, *n* = 3). (**B**) The validation of the *Ts*Nfe2l binding ability to the core elements of the *Ts*ATPGD promoter. (**I**) A double-luciferase analysis of the activation ability of a *Ts*Nfe2lΔMBR-hNfe2l2MBR fusion protein to the reporter plasmid p*Ts*ATPGDp inserted with a *Ts*ATPGD promoter fragment. The data are standardized by the mean of the pGL3-basic group, expressed as mean ± S.D. An asterisk “*” indicates significant differences between groups (*p* < 0.01, *n* = 3). (**II**) A double-luciferase analysis of the effects of ML385 on *Ts*Nfe2lΔMBR-hNfe2l2MBR and hNfe2l2 on the activation of p*Ts*ATPGDp. The data are standardized by the mean of the ML385-untreated group, expressed as mean ± S.D. An asterisk “*” indicates a significant difference between the groups (*p* < 0.01, *n* = 3). (**III**) The EMSA results of the *Ts*ATPGD promoter core element (*Ts*ARE). The left figure shows the results of the normal probe (core sequence TGATACACG), and the right figure shows the results of the mutant probe (core sequence ACTTACAGC). M refers to the nucleic acid marker, which contains bands corresponding to 2000 bp, 1000 bp, 750 bp, 500 bp, 250 bp, and 100 bp, respectively, from top to bottom. The probe length is 59 bp, including two *Ts*ARE sites. **a.** A double GST-*Ts*MafK dimer *Ts*ARE complex migration band. **b.** A single GST-*Ts*MafK dimer *Ts*ARE complex migration band. **c.** A double GST-*Ts*Nfe2lMBR—GST-*Ts*MafK dimer *Ts*ARE complex migration band. **a1**, **b1,** and **c1** are the corresponding protein bands. (**C**) The 3D structure diagram of the *Ts*Nfe2l bZIP domain combined with *Ts*ARE. (**D**) An ML cladogram of *Ts*ATPGD and homologous proteins in mollusks and vertebrates and a schematic diagram of their domains. The sequences used for tree construction are *Crassostrea virginica* (XP_022325739.1), *Pecten maximus* (XP_033728500.1), *Mizuhopecten yessoensis* (XP_021350145.1), *Mytilus coruscus* (cac5423555.1), *Homo sapiens* (NP_001159694.1), *Bos taurus* (XP_024843397.1), *Mus musculus* (NP_598909.2), and *Gallus gallus* (NP_001166064.1). *Ts*ATPGD is shown in bold. (**E**) The effects of ML385 on *Ts*ATPGD expression and product synthesis in *T. squamosa* gill cells. (**I**) The changes in carnosine content in gill tissue before and after ML385 treatment. The figure on the left shows the LC-ESI-MS spectra of the untreated group, the treated group, and a 5 µg *L*-carnosine standard. A representative mass spectrum image was taken from the position, as indicated by the red arrow of chromatography. The right figure shows the change in carnosine content before and after treatment, calculated according to a standard curve and expressed as mean ± S.D. An asterisk “*” indicates a significant difference between the groups (*p* < 0.01, *n* = 3). (**II**) The expression of *Ts*Nfe2l and *Ts*ATPGD in gill cells before and after ML385 treatment. An asterisk “*” indicates that there is a significant difference between the groups (*p* < 0.01, *n* = 3). (**F**) A schematic diagram of *Ts*Nfe2l inhibiting lipid peroxidation by regulating *Ts*ATPGD expression.

Interestingly, carnosine synthase (ATPGD), which can synthesize the potent antioxidant L-carnosine, has been identified in the *Ts*Nfe2l target gene set. To investigate the relationship between *Ts*Nfe2l and lipid peroxidation, we treated gill tissue with the Nfe2l2 inhibitor ML385. Increased lipid peroxidation levels and decreased *L*-carnosine content were observed following ML385 treatment (Figure 4A,E), suggesting a critical role of *Ts*Nfe2l in regulating antioxidant defense through *Ts*ATPGD.

Furthermore, dual-luciferase reporter assays demonstrated that *Ts*Nfe2l, in complex with *Ts*MafK, activates the transcription of *Ts*ATPGD by binding to an ARE-like element in the *Ts*ATPGD promoter (Figure 4B). The EMSA assays confirmed the binding of *Ts*Nfe2l/*Ts*MafK to the identified ARE motif (TGATACAGC), including the core element of the *Ts*ATPGD promoter. Meanwhile, the binding ability disappeared when the core element was mutated (ACTTACAGC, where the mutation site is underlined), even in the presence of *Ts*MafK (Figure 4B). Phylogenetic analysis indicates that *Ts*ATPGD is a mollusk-specific carnosine synthase lacking the ATPgrasp_N domain found in vertebrate homologs (Figure 4D). After treatment with ML385 in gills, LC-MS analysis shows that carnosine content significantly decreased. Correspondingly, the expression of *Ts*ATPGD also significantly diminished, but the expression of *Ts*Nfe2l remained unaltered (Figure 4E). These findings demonstrate that *Ts*Nfe2l regulates lipid peroxidation by transcriptionally activating *Ts*ATPGD, leading to increased *L*-carnosine production and enhanced antioxidant capacity in *T. squamosa* gill tissue (Figure 4F).

## 4. Discussion

*T. squamosa* harbors symbiotic zooxanthellae, which contribute to its nutritional equilibrium while generating ROS as a byproduct of photosynthesis [6]. Suboptimal ROS generated during photosynthesis may negatively impact the redox equilibrium of the host. Interestingly, the host clam maintains redox homeostasis without apparent oxidative stress, suggesting efficient antioxidant defense mechanisms [35,36]. Our study identified *Ts*Nfe2l, a novel Nfe2l-like transcription factor, as a key regulator of this process. *Ts*Nfe2l expression was highest in the gill tissue, which is exposed to the external environment and likely experiences augmented oxidative stress. Moreover, growing evidence supports the notion that the initial assimilation of inorganic carbon (C*i*), oxygen exchange, osmic regulation, and nutrient uptake also depend on the gill’s physiological activities [2,37,38,39]. Thus, oxygen consumption itself in various processes inevitably leads to some degree of unwanted ROS accumulation, and this Nfe2-like localization is consistent with the roles of Nfe2-like factors in keeping ROS homeostasis in the optimum against environmental stressors.

Comparative analysis reveals that *Ts*Nfe2l shares structural and functional similarities with mammalian Nfe2l1, including the presence of an *N*-terminal Neh1-like domain and a conserved bZIP domain [40]. However, *Ts*Nfe2l lacks the transcriptional activation domains found in Nfe2l1 and Nfe2l2, suggesting a distinct regulatory mechanism [40,41]. The absence of a Keap1-like protein in *T. squamosa* may also contribute to the differential regulation of *Ts*Nfe2l relative to its mammalian counterparts [15,17,40,42]. The bZIP domain, located at the *C*-terminus of bZIP-NFE2L family members, is highly conserved across species, including *Ts*Nfe2l. This conserved domain contains the DNA-binding motif, which is essential for recognizing ARE sequences [43]. Logically, the structural similarity of the bZIP domain between *Ts*Nfe2l and its mammalian counterparts likely enables *Ts*Nfe2l to bind to AREs with similar affinity and specificity. Moreover, the requirement for a Maf protein partner for efficient DNA-binding, a conserved feature of the bZIP-NFE2L family, is also maintained in *Ts*Nfe2l.

To identify potential target genes, we employed FIMO to predict *Ts*Nfe2l binding sites within 2000 bp upstream of the *T. squamosa* gene start sites. Gene ontology (GO) enrichment analysis of these predicted targets reveals *Ts*Nfe2l involvement in diverse biological processes, including neural regulation, development, and reproduction, in addition to the expected antioxidant functions [9,16,17]. This broad spectrum of potential target genes suggests that *Ts*Nfe2l may possess pleiotropic regulatory functions, similar to its mammalian counterparts Nfe2l1 and Nfe2l2 [44,45]. Nevertheless, motif-based prediction is not always accurate, especially in non-model unique bivalve species that lack substantial investigation. In conjunction with the expression correlation study, 131 predicted intersection genes were identified, and partial genes were functionally validated, supporting the successful finding of unknown transcriptional target genes by using our overlapping screening strategies. In particular, a couple of enzymes directly involved in antioxidant defense and cellular protection including carnosine synthase (*Ts*ATPGD), glutathione peroxidase (*TsGPx*), glutamine synthase (*TsGS*), NAD(P)H quinone oxidoreductase 1 (*TsNQO1*), acetaldehyde dehydrogenase (*TsAD*), and DNA synthase κ (*TsDNApolκ*) were identified, highlighting the essential role of Nfe2l in maintaining redox homeostasis across organisms from bivalves to humans [46,47,48]. Moreover, the genes *TsHSP*, *TsTNFαIP3*, *TsFGF*, and *TsPXMP* represent broader cellular functions such as stress response, inflammation, growth, and metabolism [49,50,51,52,53,54,55,56,57,58], strongly suggestive of *Ts*Nfe2l physiological importance in regulating a diverse set of target genes beyond its functions in antioxidant defense (Figure 3E).

Carnosine synthase (*Ts*ATPGD) catalyzes the synthesis of *L*-carnosine from *β*-alanine and *L*-histidine with the consumption of ATP [58,59]. *L*-carnosine and its analogs possess antioxidant properties and are found in mammalian muscle and nervous tissue, as well as in the adductor muscle of certain bivalves [59,60,61]. While the functional roles of carnosine synthase have been explored in mammals, its regulation in bivalves remains largely obscure. Our study reveals that *Ts*Nfe2l regulated *Ts*ATPGD’s transcription, which helps fill a major gap in the regulation of carnosine synthesis. Through the *Ts*Nfe2l-*Ts*ATPGD pathway, giant clams counteract the impact of lipid peroxidation by synthesizing *L*-carnosine, thereby maintaining redox equilibrium in the gill. Therefore, *L*-carnosine can not only augment the antioxidant properties of *T. squamosa* but also boost lipid metabolism and meat quality, thus improving growth parameters. Of note, under the condition of high-density and intensive culture, *L*-carnosine could effectively enhance immune function and the defense against certain diseases, which should improve breeding yields and promote the development of the marine product-based economy. In terms of application prospects, *L*-carnosine is envisioned to have strong potential for *T. squamosa* culture and other allied mariculture. However, it is worth noting that the inhibition of *Ts*Nfe2l did not completely reduce the content of *L*-carnosine (Figure 4E), suggesting that *T. squamosa* may possess other endogenous pathways controlling the synthesis of *L*-carnosine. A deepened understanding of *T. squamosa* and further investigating these pathways can help us explore the rational use of *L*-carnosine and unlock its potential.

In summary, *Ts*Nfe2l dictates antioxidant defense by activating the transcription of target genes. The identification of *Ts*ATPGD as a downstream target of *Ts*Nfe2l in this study has added some fresh mechanistic nuances to antioxidant defense strategies deployed in *T. squamosa*. The resultant elevation in *L*-carnosine production likely contributes to the host’s capacity to resolve oxidative stress. Therefore, the *Ts*Nfe2l-*Ts*ATPGD axis represents a novel facet of molecular mechanisms for maintaining the redox homeostasis necessary for photosymbiosis in this organism. In future, exploring the interactions between *Ts*Nfe2l and other antioxidant pathways would further advance our understanding of the redox regulatory networks in *T. squamosa* in ecologically relevant contexts such as coral reef bleaching. This work also provides insights into the practical protection of this rare and unique species and informs strategies for the artificial construction and restoration of certain island and reef ecosystems.

## Figures and Tables

**Table 1:** The primers used in this study (Notation: the giant clam genome project has been deposited at the NCBI under the BioProject number PRJNA673920).

Target Segment	Purpose	Sequence (5′→3′)
*Ts*Nfe2l	PCR amplification	ACCACACAAAGAAGAACGCCAAG
		TCACTCATACTTCCGATCATCATGTCT
	pGEX4T-1-TsNfe2l construction	CCGCGTGGATCCCCGGAATTCATGTTGAAACAATATTTCACAGATGGT
		GTCACGATGCGGCCGCTCGAGTCACTCATACTTCCGATCATCATGT
	pcDNA3.1-TsNfe2l construction	GCCACCATGTTGAAACAATATTTCACAGATGGT
		CTCATACTTCCGATCATCATGTCTTT
		TAGTCCAGTGTGGTGGAATTCGCCACCATGTTGAAACAATATTTC
		GAAGGGCCCTCTAGACTCGAGCTCATACTTCCGATCATCATGTCTTT
	qPCR	CGCAGCTCAGAATTGTCGGA
		AACGGGACGGGTCGTAAGG
*Ts*MafK	PCR amplification	ATGCAGCAGTCGAGTATGAAGC
		TGGTGACGAAGTGAAGATGAAAT
	pGEX4T-1-TsMafK construction	CCGCGTGGATCCCCGGAATTCATGCAGCAGTCGAGTATGAAGCA
		GTCACGATGCGGCCGCTCGAGTTACACCCGCGGCTCTGG
	qPCR	CTTCGCAATGAGGTGGATAGATTA
		GGAAATCATGGGTGGCTTGG
ARE	EMSA probe	GGCCTAACTGGCCGGTACC
		GGTGGCTTTACCAACAGTACCGG
*Ts*Nfe2lΔMBR-*H*Nfe2l2MBR	plasmid construction	TAGTCCAGTGTGGTGGAATTCGCCACCATGTTGAAACAATATTTC
		CTACAGGGAATGGAACGTTCAATTCCTGAATTCTTTTG
		GAACGTTCCATTCCCTGTAGAAAAAATCATTAA
		GAAGGGCCCTCTAGACTCGAGGTTTTTCTTAACATCTGGCTTCTTACT
*H*Nfe2l2ΔMBR-*Ts*Nfe2lMBR	plasmid construction	TAGTCCAGTGTGGTGGAATTCATGATGGACTTGGAGCTGCCG
		GTGAAAGGGATATGGAGAGCTTTTGCCCTAA
		GCTCTCCATATCCCTTTCACAATGGCACAGATAGTC
		GAAGGGCCCTCTAGACTCGAGCTCATACTTCCGATCATCATGTCTTT
*H*Nfe2l2Neh4 + 5-*Ts*Nfe2lMBR	plasmid construction	GCCACCATGCCCAAATCAGATGCTTTG
		TAGTCCAGTGTGGTGGAATTCGCCACCATGCCCAAATCA
		TGCCATTTTGGCTTCTGGACTTGGAACC
		GTCCAGAAGCCAAAATGGCAAACAGAAAAGGTAAAGGT
		GAAGGGCCCTCTAGACTCGAGCTCATACTTCCGATCATCATGTCTT
*Ts*ATPGD promoter	PCR amplification	ATTAGATATTGTGATACAGCAGACTTGC
		CATCAACAAAGTTAAAGTGCTTCCA
	plasmid construction	GCGTGCTAGCCCGGGCTCGAGATTAGATATTGTGATACAGCAGACTTGC
		CAGTACCGGAATGCCAAGCTTCATCAACAAAGTTAAAGTGCTTCCA
	EMSA probe	TTAGATATTGTGATACAGCAGACTTGCGATTAGATATTGTGATACAGCAGACTTGCGA
		TCGCAAGTCTGCTGTATCACAATATCTAATCGCAAGTCTGCTGTATCACAATATCTAA
	Mutated EMSA probe	TTAGATATTGACTTACAGCAGACTTGCGATTAGATATTGACTTACAGCAGACTTGCGA
		TCGCAAGTCTGCTGTAAGTCAATATCTAATCGCAAGTCTGCTGTAAGTCAATATCTAA
*Ts*ATPGD	qPCR	ATCTACGGGACTGGGTGAAACG
		ACTGGTTCCATATCACCTCCTCTG
*Ts*NQO1	qPCR	ATTTCCTGCGAGTACAACCA
		CAGCCTATTTCTCCCGTCA
*Ts*GPx	qPCR	AGTCATTGTGCCCGAGTGTCT
		TTGTTGCATCTGTGGGTCCTT
*Ts*TNFαP3	qPCR	GCAAAGCAGGAGGTCAAAATAAC
		CCAAATAATGTGCAGTTCGGTTC
*Ts*DNApolκ	qPCR	CAAGATGTTCTCCAGGTTCG
		CGTTGATAGTTCTGGTGCTTTT
*Ts*PMP	qPCR	GTAGTGCCATTGTTAGAGGGCC
		CTGCCATCTGTCTCCTCTTCACT
*Ts*Keap1	qPCR	TCCCAAGAAATCGAGTCGGTG
		ATACTGTTAAGCCTTTGCGTGCC
*Ts*GAPDH	qPCR	CTGGTATGGCTTTCCGAGTACCT
		TGCTGCTGTGCTCGTCTCC

## Data Availability

The original contributions presented in this study are included in the article/Supplementary Materials. Further inquiries can be directed to the corresponding author(s).

## Supplementary Materials

The following supporting information can be downloaded at: https://www.mdpi.com/article/10.3390/antiox13111351/s1, Figure S1: Specific evolutionary relationship and conserved motif of *Ts*Nfe2l and homologous proteins.; Figure S2: Chromosome distribution of members of bZIP-NFE2L family in several mollusc species and vertebrates; Figure S3: Genetic structure of members of bZIP-NFE2L family in several mollusc species and vertebrates.

## Abbreviations

CNC-bZIP	Cap’n’collar basic leucine zipper;
bZIP-NFE2L	Basic leucine zipper nuclear factor, erythroid-derived 2-like;
Nrf	Nuclear factor, erythroid 2 like;
BACH	Bric-a-brac domain and cap’n’collar homolog;
sMaf	Small musculoaponeurotic fibrosarcoma oncogene homolog;
Keap1	Kelch-like ECH associated protein 1;
cDNA	Complementary deoxyribonucleic acid;
PCR	Polymerase chain reaction;
ORF	Open reading frame;
GAPDH	Glyceraldehyde-3-phosphate dehydrogenase;
qPCR	Quantitative real-time polymerase chain reaction;
EMSA	Electrophoretic mobility shift assay;
GO	Gene ontology;
MDA	Malondialdehyde;
LC-ESI-MS	Liquid chromatogram-electron spray ionization-mass spectrometer.
