# Supplementary material for: Carnosine Synthase (TsATPGD) Alleviates Lipid Peroxidation Under Transcriptional Control by an Nfe2-like Gene in Tridacna Squamosa
Source: Antioxidants (Basel). 2024 Nov 4;13(11):1351. doi: 10.3390/antiox13111351 (PMC11591149; doi:10.3390/antiox13111351)
Supplement: Supplementary file 1 [file antioxidants-13-01351-s001.zip › antioxidants-3202248-supplementary.pdf]

Figure S1

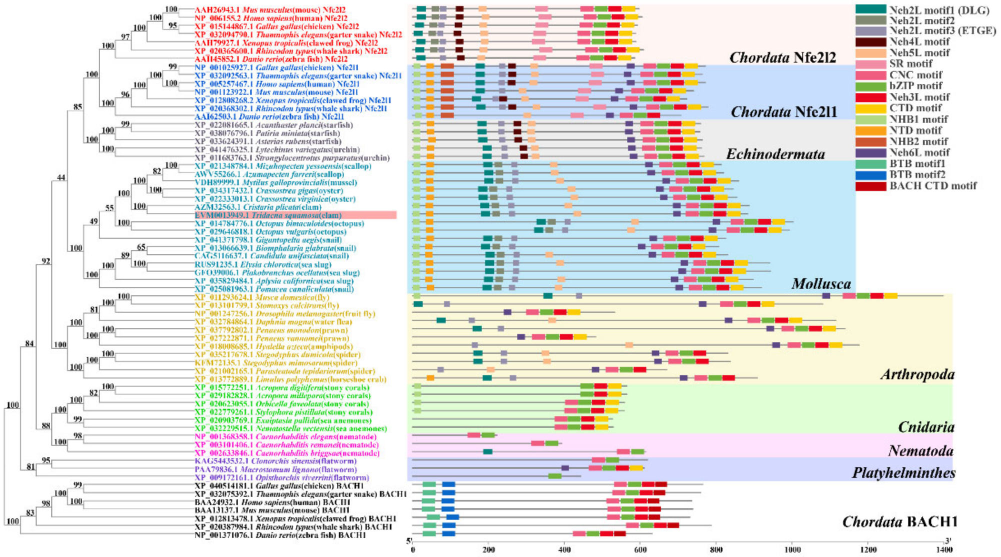

Figure S1. Specific evolutionary relationship and conserved motif of *Ts*Nfe2l and homologous proteins.

Figure S2

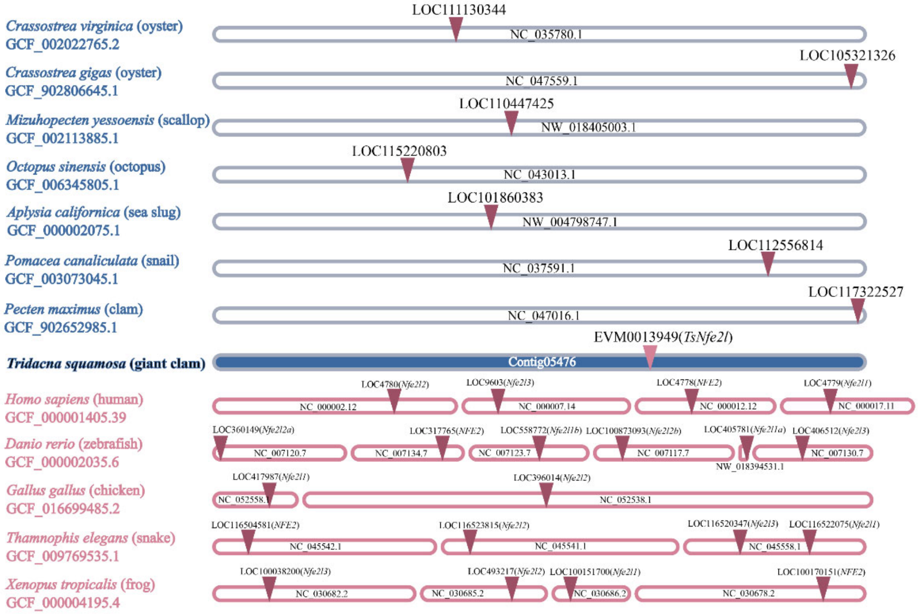

Figure S2. Chromosome distribution of members of bZIP-NFE2L family in several mollusc species and vertebrates.

Figure S3

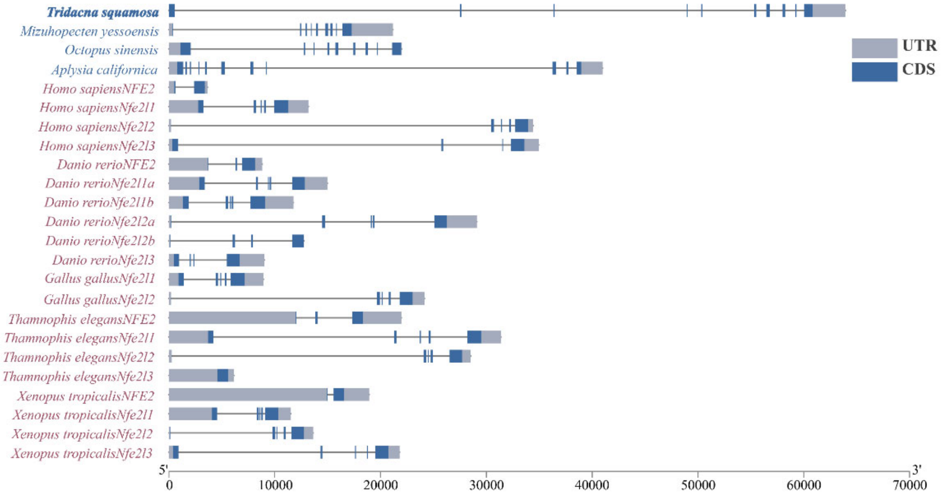

Figure S3. Genetic structure of members of bZIP-NFE2L family in several mollusc species and vertebrates.
